# Pretreatment Sarcopenia and MRI-Based Radiomics to Predict the Response of Neoadjuvant Chemotherapy in Triple-Negative Breast Cancer

**DOI:** 10.3390/bioengineering11070663

**Published:** 2024-06-28

**Authors:** Jiamin Guo, Wenjun Meng, Qian Li, Yichen Zheng, Hongkun Yin, Ying Liu, Shuang Zhao, Ji Ma

**Affiliations:** 1 Division of Abdominal Tumor Multimodality Treatment, Cancer Center, West China Hospital, Sichuan University, No. 37 Guoxue Alley, Chengdu 610041, China; 2022224025530@stu.scu.edu.cn (J.G.); zyichenscu@163.com (Y.Z.); 2 Department of Biotherapy, Cancer Center, West China Hospital, Sichuan University, No. 37 Guoxue Alley, Chengdu 610041, China; mwj1995@foxmail.com; 3 Department of Radiology, West China Hospital, Sichuan University, No. 37 Guoxue Alley, Chengdu 610041, China; lq19971129@126.com (Q.L.); ly0283@yeah.net (Y.L.); 4 Infervision Medical Technology Co., Ltd., No. 62 East Fourth Ring Middle Road, Chaoyang District, Beijing 100025, China; yinhk1985@gmail.com

**Keywords:** triple-negative breast cancer, sarcopenia, neoadjuvant chemotherapy, radiomics, Miller–Payne, pathological complete response

## Abstract

The association between sarcopenia and the effectiveness of neoadjuvant chemotherapy (NAC) in triple-negative breast cancer (TNBC) remains uncertain. This study aims to examine the potential of sarcopenia as a predictive factor for the response to NAC in TNBC, and to assess whether its combination with MRI radiomic signatures can improve the predictive accuracy. We collected clinical and pathological information, as well as pretreatment breast MRI and abdominal CT images, of 121 patients with TNBC who underwent NAC at our hospital between January 2012 and September 2021. The presence of pretreatment sarcopenia was assessed using the L3 skeletal muscle index. Clinical models were constructed based on independent risk factors identified by univariate regression analysis. Radiomics data were extracted on breast MRI images and the radiomics prediction models were constructed. We integrated independent risk factors and radiomic features to build the combined models. The results of this study demonstrated that sarcopenia is an independent predictive factor for NAC efficacy in TNBC. The combination of sarcopenia and MRI radiomic signatures can further improve predictive performance.

## 1. Introduction

Triple-negative breast cancer (TNBC), which lacks hormone receptors and human epidermal growth factor receptors 2 (HER-2), accounts for 15 to 20 percent of breast cancer (BC) subtypes [1]. The recurrence rate of TNBC is 30–40%, and studies showed that the 5-year relative survival rate for advanced TNBC was only 14% [2,3]. However, TNBC is remarkably more sensitive to chemotherapy compared to other BC subtypes [4]. For the majority of TNBC patients, sequential neoadjuvant chemotherapy (NAC) based on anthracycline and taxane agents is the standard treatment approach [5]. NAC has the potential to lower cancer staging, decrease metastasis, evaluate drug sensitivity, and improve the likelihood of breast-conserving treatment [6].

Due to the high heterogeneity of TNBC, different patients may achieve varying responses to NAC. The Miller–Payne (MP) system is currently the most commonly used pathological assessment system, and it is used to evaluate the reduction in primary tumor cells after NAC [7]. Patients with an MP grade 5 and ypN0 after NAC are recognized as having achieved pathological complete response (pCR), which indicates highly favorable disease-free survival and overall survival [8]. Hence, pCR can serve as an early clinical endpoint that substitutes for long-term survival. However, these efficacy assessment criteria rely on postoperative pathological specimens. There is still no standardized method for predicting NAC efficacy prior to therapy. This has led many patients who are not responsive to chemotherapy to receive inappropriate treatments and delays to the optimal time for surgical removal.

Sarcopenia is a widely studied body composition parameter in oncology [9]. Current studies have demonstrated that BC patients with sarcopenia have a higher overall risk of death compared to those without it, due to complications, frailty, and metabolic dysfunction [10]. At the same time, patients with sarcopenia have shown an increased level of chemotherapy toxicity [11]. Furthermore, sarcopenia is positively correlated with pro-inflammatory cytokines, such as fibrinogen, C-reactive protein, TNF-α, and IL-6, which can promote BC progression [11]. Although many studies have associated sarcopenia with several poor clinical outcomes, there is a lack of high-quality prospective cohort studies in the field to validate these conclusions [12]. Moreover, the correlation between sarcopenia and response to NAC in BC is so far unknown.

MRI, using morphological and multiple functional parameters, has shown the potential for predicting therapeutic responses [13,14,15]. MRI is considered the most sensitive imaging modality for evaluating BC treatment response to NAC [16]. Additionally, with advancements in the bioinformatics field, new methods of analyzing medical imaging data in cancer evaluation have developed [17]. Radiomics is a rapidly rising field. It enables extracting and analyzing the advanced and quantitative imaging features. By deep mining the imaging features, it is possible to determine the relationship between these features and the underlying pathophysiology of the tumor [18]. Several studies have suggested that MRI-based radiomics has the potential to predict the treatment response to NAC. One study constructed radiomics-only models to predict pCR in TNBC. The AUCs for the radiomics-only models were 0.71 and 0.73, respectively. After incorporating genomics, the radiogenomic model had a higher predictive power than the radiomics-only model (*p* = 0.04), with a corresponding AUC of 0.87 (0.73–0.91) [19]. The Multi-Layer Perception (MLP) constructed by Huang et al. predicted TNBC pCR with AUCs of 0.837–0.901 in the external validation set [20]. Additionally, we have summarized recent studies that constructed radiomic models to predict the efficacy of NAC in TNBC [21,22,23,24] (Supplementary Table S1). However, to our knowledge, the NAC efficacy prediction models in TNBC using pretreatment sarcopenia and MRI-based radiomics have not been reported.

Therefore, we hypothesized that sarcopenia might be an important pre-NAC predictor and that combining it with breast MRI radiomics might develop better prediction models.

## 2. Materials and Methods

### 2.1. Patients

**Figure 1:**
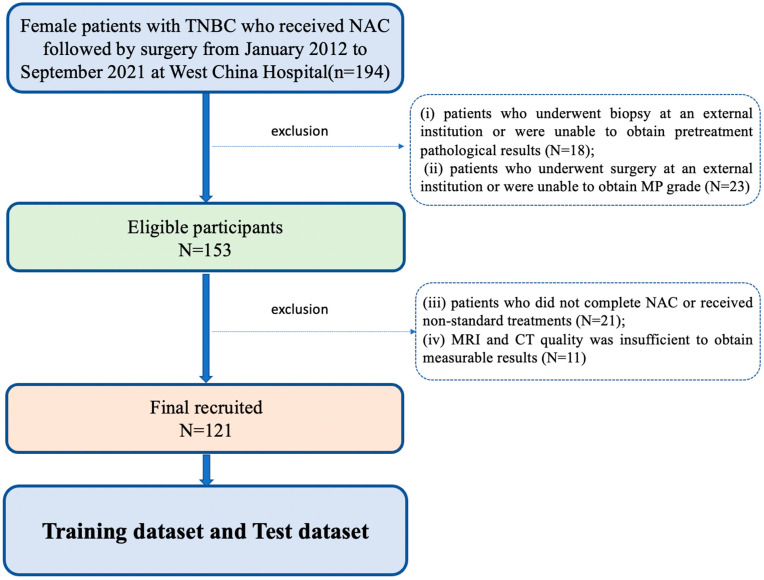
Flow chart of patient selection. TNBC: triple-negative breast cancer, NAC: neoadjuvant chemotherapy, MP: Miller–Payne.

This retrospective study was approved by the Ethical Committee of West China Hospital, and the request for informed consent was waived. Patients enrolled in this study included female patients with TNBC who underwent NAC treatment followed by surgery between January 2012 and September 2021 in our hospital. All patients received the standard NAC regimen recommended by the National Comprehensive Cancer Network (NCCN) guidelines. The main regimens were based on anthracyclines, taxanes, with or without alkylating agents, and for some high-risk patients, the platinum-containing chemotherapy regimens were employed [25]. The following were the inclusion criteria: (i) patients who had primary TNBC confirmed by biopsy and without distant metastasis; (ii) patients who underwent complete NAC therapy; (iii) patients who underwent surgery at our hospital after completion of NAC, with postoperative pathological examination including MP grade; (iv) breast MRI performed before treatment, including T1C sequence; (v) pretreatment CT scan was performed, covering the level of L3 vertebra. The following were the exclusion criteria: (i) patients who underwent biopsy at an external institution or were unable to obtain pretreatment pathological results; (ii) patients who did not complete NAC or received non-standard treatments; (iii) patients who underwent surgery at an external institution or were unable to obtain MP grade; (iv) MRI and CT quality was insufficient to obtain measurable results. Eventually, 121 TNBC patients were enrolled in our study, as shown in Figure 1.

### 2.2. Clinical Feature Collection

The clinical variables of each patient were collected, including age, weight, body mass index (BMI), menopausal status, clinical tumor staging (T-stage and N-stage), Ki-67 index, tumor markers (CEA and CA-153), and treatment parameters (NAC regimens and cycles). The preoperative TNM staging was assessed using CT, MRI, and/or whole-body PET/CT, according to the eighth edition of the American Joint Committee on Cancer (AJCC) staging system.

### 2.3. Pathological Examination and Response to Treatment

Histopathological assessment was conducted independently by two pathologists with more than 5 years of experience in breast pathology and unaware of the MRI results. Discrepancies were reviewed by the two pathologists and agreement was reached through discussion. According to the NCCN guidelines, this study defined an MP grade of 1–3 (MP-Low) after NAC as a poor treatment response, while MP grades 4 and 5 (MP-High) were defined as a better treatment response. Specifically, an MP grade 5 (primary tumor bed without invasive cancer cells but may contain ductal carcinoma in situ) with ypN0 group corresponded to pCR, and it was defined as the absence of residual invasive carcinoma in both the breast and axillary lymph nodes, while carcinoma in situ was permitted in certain cases (ypT0/Tis ypN0). After NAC, patients with residual lesions in the breast or axilla, or both, were considered to have not achieved pCR, known as non-pCR.

### 2.4. Body Composition Quantification and Sarcopenia Assessment

Patient body composition quantification characteristics at baseline were collected, which included SMA, SMI, subcutaneous fat area (SFA), subcutaneous fat thickness (SFT), mean muscle attenuation, and sarcopenia status. 

CT data were acquired prior to NAC. The CT images of each patient were utilized to measure skeletal muscle and subcutaneous fat at the level of the L3 vertebra (Reference to Supplementary File S1—CT Scanning Parameters). Skeletal muscle and subcutaneous fat boundaries were manually delineated by two trained observers also using the uAI Research Platform. Subsequently, cross-sectional areas were automatically computed by summing the tissue pixels and multiplying by the pixel area. Additionally, abdominal SFT was measured on the umbilical level line ± 5 cm from the navel. The measurements were averaged between the left and right sides. SMI was calculated by normalizing the L3 muscle cross-sectional area to height. According to the European Consensus, sarcopenia was recognized as L3 SMI < 39 cm^2^/m^2^ for females [26]. Mean muscle attenuation in Hounsfield units indirectly measured fat infiltration in muscles at the L3 level. We measured muscle area, adipose area, and muscle attenuation on CT plain images.

### 2.5. MRI Protocol and Image Segmentation

Each patient underwent breast MR examination before biopsy and within 1 to 2 weeks before NAC. Breast MRI was carried out on a 3.0 T scanner using a dedicated breast coil and patients were kept in the prone position. All protocols adhered to international guidelines [27] (Reference to Supplementary File S2—MRI Scanning Parameters for specific protocols). Each TNBC lesion was identified in axial contrast-enhanced T1-weighted imaging (T1WI) sequences. Regions of interest (ROIs) were manually delineated by two radiologists with over 10 years of experience in breast imaging using the commercial artificial intelligence platform uAI Research Platform (United Imaging Intelligence, Shanghai, China). In cases of uncertain boundaries, the consultation was sought from another radiologist with 20 years of breast imaging experience to make the final decision. The radiologists were blinded to all pathological and clinical findings.

### 2.6. Radiomics Feature Extraction

The MR images were resampled to the 1 mm × 1 mm × 1 mm voxel size by the B-spline surface construction, and the low-frequency intensity non-uniformity was also corrected with the N4 bias field correction algorithm [28]. The PyRadiomics package (version 3.0.1) was used for the automated extraction of radiomics features from the manually labeled ROIs following the latest recommendations of the image biomarker standardization initiative with the bin size fixed to 32 [29]. Finally, a total of 14 shape radiomics features, 342 first-order statistics radiomics features, and 1387 high-level texture radiomics features were extracted from each ROI, respectively (Supplementary File S3—Radiomics Features).

In order to prevent overfitting and reduce the complexity of the radiomics model, the radiomics features were selected before model development. Firstly, the inter-observer reliability of the features was assessed through the intraclass correlation coefficient (ICC) test and only the ones with an ICC > 0.8 were retained for subsequent analysis. Then, the difference in the radiomics features between the two groups was evaluated by the Mann–Whitney U test and the features that exhibited significant differences (*p* < 0.05) were kept. Then, the Pearson correlation coefficient analysis was applied for the evaluation of feature redundancy. If the correlation between two radiomics features was higher than 0.95, the one with a larger *p*-value in the previous Mann–Whitney U test was excluded. Finally, the Least Absolute Shrinkage and Selection Operator (LASSO) regression analysis was used to find out the most critical radiomic features (Supplementary File S4—LASSO parameters), and the penalty parameter was determined via 10-fold cross-validation according to the “minimum mean-squared error” rule. The flowchart of feature selection is shown in Supplementary Figure S1.

### 2.7. Model Development and Evaluation

Univariate logistic regression analysis was used to identify the significant clinical and body composition variables with MP grade or pCR. Only the variables that showed noteworthy association (*p* < 0.05) with the NAC prognosis were considered risk factors and were used to construct clinical prediction models.

Based on the selected radiomics features, the radiomics model was first developed in this study. The enrolled patients were randomly split into five folds with roughly equal sample sizes. One fold was held out for validation, with the remaining four folds used for model development, and this process was repeated for each of the five folds [30]. Supplementary Figure S2 demonstrates the flowchart of five-fold cross-validation. A total of 6 well-known machine learning classifiers were applied for model construction, including the Linear Discriminant Analysis (LDA), Logistic Regression (LR), Multi-Layer perceptron (MLP), Naïve Bayes Bernoulli (NBB), Random Forest (RF), and Support Vector Machine (SVM) classifiers. Machine learning algorithm parameters for this study are detailed in the Supplementary File S5—Machine learning classifier parameters.

The combined models were established by integrating independent clinical risk features with radiomic features. In brief, clinical risk factors selected through univariate regression analysis were used alongside the screened radiomic features as inputs for the machine learning classifier. Five-fold cross-validation was employed to develop and validate the combined models. The data splitting and the machine learning classifiers were exactly the same as the ones used for the development of the radiomics models.

**Figure 2:**
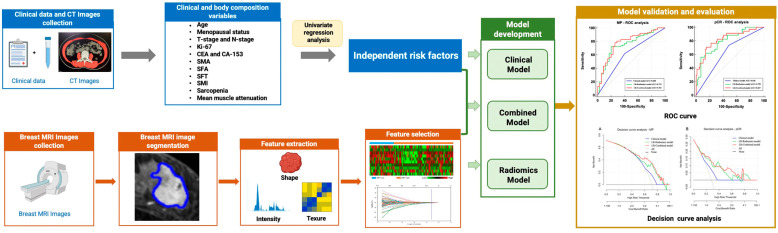
Framework and flowchart of the study. The workflow included clinical and body composition variables collection, independent risk factors identification, pre-NAC MRI images collection and ROI delineation, image feature extraction, machine learning prediction models construction, validation, and evaluation. SMA: skeletal muscle area, SMI: skeletal muscle index, SFA: subcutaneous fat area, SFT: subcutaneous fat thickness.

The discrimination capability of the predictive models was evaluated by the receiver operating characteristics (ROC) analysis and compared by assessing the area under the curve (AUC). The ROC curve was generated by calculating and plotting a series of true positive rates and false positive rates across a variation in the decision threshold (typically, from 0 to 1). The optimal threshold values of the radiomics and the combined models were determined by maximizing Youden’s J statistic. In addition, the sensitivity, specificity, positive predictive value (PPV), and negative predictive value (NPV) of each model were also computed, and the optimal cut-off point was determined by maximizing the Youden index. The assessment of the goodness-of-fit of each model was performed by calibration analysis, and the Hosmer–Lemeshow test was used for the evaluation of consistency between the model predicted probability and actual observed rate [31]. In addition, the decision curve analysis (DCA) was also used to evaluate and compare the clinical usefulness of different models by calculating the net benefits across a range of reasonable threshold probabilities [32]. The development and validation of the predictive models were performed by using the InferScholar platform (version 3.5, Infervision, East Brunswick, NJ, USA), as shown in Figure 2.

### 2.8. Statistical Analysis

All statistical analysis was conducted using the SPSS (Version 21.0; IBM, Armonk, NY, USA) software. Descriptive statistics were reported as mean ± standard deviation. The *t*-test or Mann–Whitney U test was used for group comparisons of the quantitative variables, and the χ^2^ tests or Fisher’s test was used for the qualitative variables. Differences between the two AUCs were compared by the DeLong test. All statistical tests were two-sided, and a *p*-value less than 0.05 was considered statistically significant.

## 3. Results

### 3.1. Characteristics of the Patients

A total of 121 pre-NAC T1C sequence MRI images and CT plain images were obtained. Patients were excluded due to poor quality or absence of pre-NAC MRI or CT images (*n* = 11), inadequate clinical information (*n* = 18), missing information on treatment response (*n* = 23), or incomplete NAC therapy (*n* = 21) (Figure 1). In the dataset, 71.1% of patients (86/121) were identified as MP-High and 28.9% of patients (35/121) as MP-Low. In total, 28.1% of patients (34/121) achieved pCR after NAC, and 71.9% of patients (87/121) had non-pCR. 

Among these 121 TNBC patients (mean age 48.7, IQR 37.7–59.7), 44.6% of patients suffered from sarcopenia before NAC (54/121). In the MP-Low cohort, 60% of patients (21/35) had pretreatment sarcopenia, whereas in the MP-High cohort, 38.4% of patients (33/86) suffered sarcopenia prior to NAC. Only 16.7% of patients (9/54) with sarcopenia achieved pCR after NAC, while more than half of patients with sarcopenia (45/54) were non-pCR. As shown in Table 1, there was a significant difference in sarcopenia prevalence between the MP-Low patients and MP-High patients, as well as the non-pCR patients and pCR patients (*p* < 0.05). Other baseline characteristics of patients did not demonstrate statistical differences between the MP-High/Low groups and the pCR/non-pCR groups.

### 3.2. Clinical and Body Composition Variables Associated with NAC Efficacy

A total of 14 clinical and body composition variables were collected and further analyzed by univariate regression analysis. As presented in Table 2, only the sarcopenia had a significant correlation with the MP grade (odds ratio [OR], 0.4151 (95% CI: 0.1858–0.9274); *p* < 0.05). Similarly, sarcopenia also showed a correlation with pCR/non-pCR in the dataset ([OR], 0.3360 (95% CI: 0.1407–0.8022); *p* < 0.05).

### 3.3. Radiomics Feature Selection

There were 56 MP-associated radiomics features and 73 pCR-associated radiomics features retained for the LASSO regression analysis after the ICC analysis, univariate analysis, and Pearson correlation analysis, respectively. As shown in Supplementary Figure S3, a total of 10 MP-associated radiomics features and 9 pCR-associated radiomics features with non-zero coefficients were finally selected for model development under the optimal tuning parameters (lambda = 0.0754 and 0.0958 for MP and pCR, respectively). The heatmaps of the selected MP-associated and pCR-associated radiomics features were plotted against the standardized feature values (Supplementary Figure S4). The weights and biological significance of the radiomics features were shown in the Supplementary File S6—Feature weights and biological meaning.

### 3.4. Performance Evaluation of MP-Low/High Prediction Models

**Figure 3:**
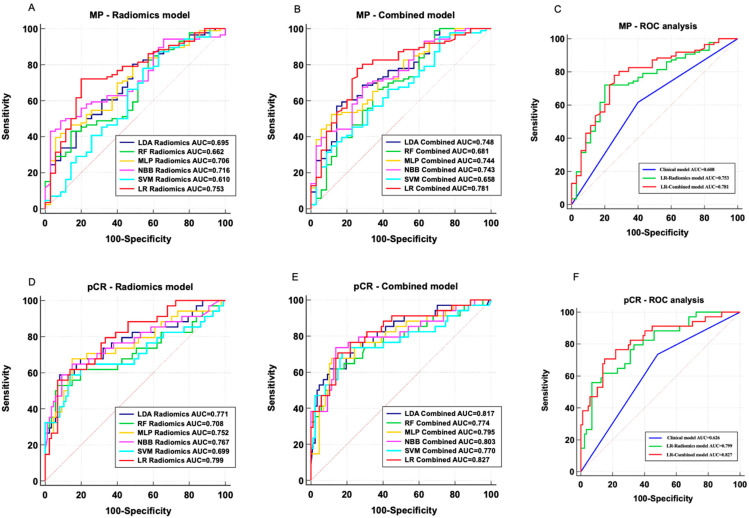
Performance of each predictive model in the MP-Low/High and pCR/non-pCR classification. (**A**) ROC analysis of the radiomics models in the MP validation dataset. (**B**) ROC analysis of the combined models in the MP validation dataset. (**C**) ROC analysis of the clinical model, LR radiomics model, and LR combined model in the MP validation dataset. (**D**) ROC analysis of the radiomics models in the pCR validation dataset. (**E**) ROC analysis of the combined models in the pCR validation dataset. (**F**) ROC analysis of the clinical model, LR radiomics model, and LR combined model in the pCR validation dataset. AUC: area under curve, LDA: Linear Discriminant Analysis, RF: Random Forest, MLP: Multi-Layer perceptron, NBB: Naïve Bayes Bernoulli, SVM: Support Vector Machine, LR: Logistic Regression.

The AUC, sensitivity, specificity, PPV, and NPV of each model in the validation dataset for the MP-Low/High prediction models are summarized in Table 3. The AUC value of the clinical model based on sarcopenia was 0.608 (95% CI: 0.515–0.696). The radiomics models used LDA, RF, MLP, NBB, SVM, and LR, with AUCs achieving 0.695 (95% CI: 0.605–0.775), 0.662 (95% CI: 0.571–0.746), 0.706 (95% CI: 0.616–0.785), 0.716 (95% CI: 0.627–0.794), 0.610 (95% CI: 0.517–0.697), and 0.753 (95% CI: 0.667–0.827), respectively (Figure 3A). After incorporating sarcopenia, the AUCs of the LDA, RF, MLP, NBB, SVM, and LR classifier-based combined models were improved to 0.748 (95% CI: 0.660–0.822), 0.681 (95% CI: 0.590–0.763), 0.744 (95% CI: 0.657–0.819), 0.743 (95% CI: 0.656–0.818), 0.658 (95% CI: 0.566–0.742), and 0.781 (95% CI: 0.697–0.851), respectively (Figure 3B). The LR classifier-based models showed the best discrimination capability in both radiomics models and combined models. The clinical model, LR radiomics model, and LR combined model performance are illustrated in Figure 3C.

### 3.5. Performance Evaluation of pCR/Non-pCR Prediction Models

Similarly, the AUC, sensitivity, specificity, PPV, and NPV of the pCR/non-pCR prediction models were evaluated and compared on the validation dataset (Table 4). The sarcopenia-based clinical model exhibited an AUC of 0.626 (95% CI: 0.534–0.713). The LDA, RF, MLP, NBB, SVM, and LR classifier-based radiomics models had achieved AUCs of 0.771 (95% CI: 0.686–0.842), 0.708 (95% CI: 0.619–0.787), 0.752 (95% CI: 0.665–0.826), 0.767 (95% CI: 0.682–0.839), 0.699 (95% CI: 0.609–0.779), and 0.799 (95% CI: 0.716–0.866), respectively (Figure 3D). The combined model used these machine learning algorithms (LDA, RF, MLP, NBB, SVM, and LR), with AUCs reaching 0.817 (95% CI: 0.737–0.882), 0.774 (95% CI: 0.689–0.845), 0.795 (95% CI: 0.712–0.863), 0.803 (95% CI: 0.721–0.870), 0.770 (95% CI: 0.684–0.841), and 0.827 (95% CI: 0.747–0.889), respectively (Figure 3E). Regardless of the radiomics models or the combined models, the LR classifier still demonstrated the best classification performance. Therefore, the best-performing LR radiomics and LR combined models were selected for comparison with the clinical model, as shown in Figure 3F.

### 3.6. Important Model Features

**Figure 4:**
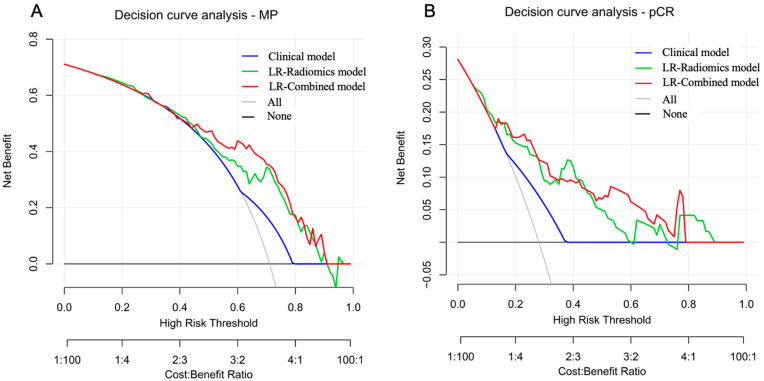
Decision curve analysis of the predictive models in the MP-Low/High and pCR/non-pCR validation dataset. (**A**) Calibration curves of the clinical model, LR radiomics model, and LR combined model for MP classification. (**B**) Calibration curves of the clinical model, LR radiomics model, and LR combined model for pCR classification.

The robustness of each model was also evaluated by comparing the AUCs between the training and validation datasets. Only the LR classifier-based models showed good robustness with no remarkable difference in AUCs between the training dataset and the validation dataset (*p* > 0.05) (Supplementary Tables S2 and S3). In addition, the Hosmer–Lemeshow test in the validation dataset suggested all models had good calibration, as the non-significant statistic of all models was larger than 0.05 (Supplementary Data S1). The decision curve analysis in the validation dataset demonstrated that the predictive models had higher net benefits across the majority of threshold probabilities than the treat-all and treat-none policies (Figure 4).

## 4. Discussion

NAC provides surgical opportunities for patients with locally advanced TNBC, reduces the clinical staging of the tumor, and increases the chances of breast-conserving surgery. However, 10–35% of TNBC patients have poor outcomes from NAC, and some even experience disease progression during the NAC period [33]. Accurately and non-invasively identifying the beneficiary group of NAC is a major challenge in this field. In this study, we have confirmed that sarcopenia was a factor in predicting the efficacy of NAC in TNBC. In the validation set, the performance of the radiomics model in predicting both MP-Low/High and pCR/non-pCR was superior to that of the clinical model. When combined with sarcopenia, the efficacy in predicting NAC MP classification and pCR further improved, with AUCs of 0.781 and 0.827, respectively.

Low muscle mass or sarcopenia has been proven to be associated with more severe chemotherapy toxicity, poorer treatment outcomes, and tumor progression time (TTP) in BC patients [34]. Moreover, BC patients with sarcopenia may face a notably higher risk of death compared to those without sarcopenia [35]. Our findings demonstrated the same trend, whereby sarcopenia served as an independent predictive factor for NAC efficacy, regardless of predicting MP-Low/High or pCR/non-pCR. The information about muscle mass obtained from the L3 vertebra on CT could be a meaningful biomarker for TNBC prognosis. TNBC patients with sarcopenia achieved lower MP grades and pCR rates after NAC compared to those without sarcopenia at baseline. The potential mechanisms are very complex. Firstly, the primary characteristic of sarcopenia was muscle mass, which was the consequence of an imbalance in the protein synthesis and degradation pathways. One study suggested that this phenomenon arose due to an increase in the expression of myostatin, leading to impaired protein synthesis and diminished satellite cell function [36]. Secondly, more evidence indicated that sarcopenia was related to immune pathways and inflammation. Previous studies revealed that the poorer muscle mass was significantly associated with the higher neutrophil-to-lymphocyte ratio and pro-inflammatory cytokines [37,38]. These were markers of systemic inflammation that could accelerate tumor progression and reduce therapeutic efficacy. Thirdly, sarcopenia was linked to protein hydrolysis cascades, such as TNF-α, which was demonstrated to promote tumor migration and was associated with a worsening prognosis in BC [38]. Finally, patients with sarcopenia had a poorer nutritional and physical status, leading to lower tolerance to chemotherapy [39]. Consideration of the above factors, as well as our findings, confirmed that sarcopenia led to a risk of a poor outcome. Therefore, the assessment of sarcopenia was proposed as part of the routine clinical evaluation in BC patients.

Several previous studies constructed models to predict the efficacy of neoadjuvant therapy (NAT) for BC using MRI radiomics features and clinical pathological variables. Liu et al. showed that the radiomics model based on multi-parametric MRI achieved an optimal AUC of 0.79 for predicting pCR. By incorporating independent clinical pathological risk factors, the AUC increased to 0.86 [14]. Chen et al. demonstrated that the radiomics model had an AUC of 0.834 in the test set, and when radiomics features were combined with the hormone status, the joint model achieved an AUC of 0.879 [40]. To date, no studies have associated baseline muscle status with radiomics to create predictive models for NAT efficacy in BC patients. Moreover, previous studies focused on various subtypes of BC, and their results often indicated that adding molecular subtype information could improve the predictive performance. TNBC lacks endocrine and targeted treatment pathways, making its efficacy harder to predict. Our study focused on TNBC patients to construct predictive models, showing significant clinical relevance. The radiomics models we established achieved AUCs of 0.753 and 0.799 for predicting MP-Low/High and pCR/non-pCR, respectively, in the test dataset, which were comparable to previous studies. Introducing sarcopenia into the models, the combined models exhibited the best performance among all the tested models, with AUCs for predicting MP and pCR rising to 0.781 and 0.827. Furthermore, the decision curve analysis indicated that, over a broad range of potential thresholds, the combined models achieved a higher net benefit in the test dataset compared to the radiomic models alone, suggesting that incorporating sarcopenia had clinical application value.

Previous research combined radiomics and muscle fat information to construct predictive models. Miao et al. utilized DLR methods combined with muscle/fat information to predict distant metastasis in BC patients. Their results further confirmed that the muscle/fat level had a significant effect on BC distant metastasis, and the joint model markedly improved the predictive performance for distant metastasis [41]. Qi et al. constructed an interpretable machine learning model to explore the relationship between sarcopenia and distant metastasis of BC, and their study showed that the skeletal muscle index (SMI/T11) was a risk factor for predicting distant metastasis in BC and it was an independent prognostic factor for distant metastasis-free survival (DMFS) and OS [42]. However, there have been no studies evaluating the relationship between sarcopenia, radiomic features, and NAC efficacy in TNBC. In our study, the use of T1C sequence MRI radiomics features might be able to obtain more detailed information about the tumor that cannot be easily detected by the naked eye. More importantly, compared to independent radiomics-based predictive models, the combination of muscle mass information with radiomics data enhanced the efficacy prediction capability. The best-performing combined model not only demonstrated good feature reproducibility but also achieved a strong predictive performance in the test dataset (AUC of MP-Low/High was 0.781, AUC of pCR/non-pCR was 0.827), enabling risk stratification of TNBC patients before NAC treatment.

This study had some limitations. Firstly, it was designed retrospectively and relied on previously collected data. Secondly, the models developed in this study were based on a single-center sample, necessitating additional evidence from multiple centers for validation. Thirdly, the ROIs in this study were manually delineated, which was time-consuming. Future research can improve this by introducing deep learning-based automatic ROI segmentation methods. Lastly, the quality of life, dietary intake, and adverse reactions during patients’ NAC were not recorded, and collecting these data could provide a more comprehensive understanding of how sarcopenia affected TNBC patients’ responses to NAC.

## 5. Conclusions

Pretreatment sarcopenia is a prediction biomarker for NAC in TNBC. The proposed combined models based on sarcopenia and MRI radiomics could be strong predictors of NAC efficacy in TNBC, leading to accurate stratification and enhancing personalized therapeutic decisions of TNBC patients. In future clinical applications, evaluating patients’ muscle status before treatment, early identification of sarcopenia, and proactive interventions may help improve the efficacy of NAC in patients with TNBC. This study still needs to be confirmed by future large-scale prospective studies.

## Figures and Tables

**Table 1:** Comparison of patients’ characteristics between the MP-Low and MP-High groups and the non-pCR and pCR groups. SMA: skeletal muscle area, SMI: skeletal muscle index, SFA: subcutaneous fat area, SFT: subcutaneous fat thickness.

Clinical Variables	MP-Low (n = 35)	MP-High (n = 86)	*p*-Value	Non-pCR (n = 87)	pCR (n = 34)	*p*-Value
Age (mean ± SD)	50.4 ± 11.1	48.1 ± 11.0	0.295	49.1 ± 11.0	47.8 ± 11.0	0.587
Weight (kg, mean ± SD)	58.5 ± 4.38	61 ± 5.87	0.308	59.7 ± 4.07	60.55 ± 6.89	0.726
BMI (kg/m^2^, mean ± SD)	24.8 ± 2.62	24.97 ± 3.06	0.897	25.43 ± 2.48	24.27 ± 3.25	0.314
Menopausal status (no/yes)	17/18	41/45	0.929	43/44	15/19	0.601
T staging (T1/T2/T3/T4)	1/13/10/11	6/42/18/20	0.431	3/38/20/26	4/17/8/5	0.149
N staging (N0/N1/N2/N3)	4/15/11/5	6/38/22/20	0.596	8/39/23/17	2/14/10/8	0.881
Ki67 (mean ± SD)	0.52 ± 0.21	0.47 ± 0.23	0.226	0.48 ± 0.23	0.48 ± 0.20	0.972
CEA (mean ± SD)	3.88 ± 11.02	3.61 ± 7.87	0.883	4.13 ± 10.38	2.56 ± 2.10	0.387
CA153 (mean ± SD)	21.83 ± 13.76	20.40 ± 12.70	0.589	21.95 ± 13.37	17.91 ± 11.63	0.128
Chemotherapy regimens (three-drug/two-drug)	8/27	25/61	0.488	27/60	6/28	0.139
Chemotherapy cycles (<6/≥6)	12/23	35/51	0.514	37/50	10/24	0.185
SMA (cm^2^, mean ± SD)	96.51 ± 11.64	100.04 ± 12.85	0.165	97.66 ± 13.41	102.50 ± 9.46	0.059
SMI (cm^2^/m^2^, mean ± SD)	39.84 ± 5.13	40.78 ± 5.12	0.367	40.31 ± 5.59	41.01 ± 3.69	0.507
SFA (cm^2^, mean ± SD)	171.38 ± 51.93	159.47 ± 57.62	0.295	164.33 ± 56.05	159.31 ± 56.75	0.662
SFT (cm, mean ± SD)	2.47 ± 0.56	2.43 ± 0.76	0.790	2.42 ± 0.64	2.52 ± 0.85	0.492
Mean muscle attenuation (HU, mean ± SD)	30.84 ± 8.55	30.94 ± 9.89	0.956	30.65 ± 9.67	31.58 ± 9.09	0.632
Sarcopenia (no/yes)	14/21	53/33	0.031	42/45	25/9	0.012

**Table 2:** Univariate regression analysis of the clinical and body composition variables. SMA: skeletal muscle area, SMI: skeletal muscle index, SFA: subcutaneous fat area, SFT: subcutaneous fat thickness.

Clinical Variables	Univariate Regression—MP			Univariate Regression—pCR		
	Odds Ratio	95% CI	*p*-Value	Odds Ratio	95% CI	*p*-Value
Age	0.9809	0.9462–1.0168	0.2929	1.0268	0.9901–1.0649	0.1539
Weight	0.7483	0.3685–1.1481	0.2323	1.1522	0.5497–1.7547	0.7355
BMI	0.9796	0.7129–1.2223	0.2577	1.0881	0.4823–1.6939	0.1567
CA153	0.9919	0.9634–1.0213	0.5863	0.9780	0.9444–1.0128	0.2125
CEA	0.9968	0.9553–1.0401	0.8816	0.9648	0.8779–1.0603	0.4566
Ki67	0.3347	0.0568–1.9723	0.2265	0.8196	0.1406–4.7792	0.8250
Menopausal status	1.0366	0.4721–2.2759	0.9286	1.5714	0.7078–3.4888	0.2667
N staging	1.2276	0.7892–1.9094	0.3630	1.0968	0.7108–1.6926	0.6763
Chemotherapy regimens	0.7230	0.2893–1.8069	0.4876	2.2119	0.8219–5.9527	0.1160
Chemotherapy cycles	0.7602	0.3349–1.7260	0.5123	0.9340	0.4180–2.0867	0.8677
T staging	0.7551	0.4961–1.1493	0.1899	0.6597	0.4277–1.0174	0.0598
Mean muscle attenuation	1.0012	0.9608–1.0433	0.9556	1.0105	0.9684–1.0544	0.6294
Sarcopenia	0.4151	0.1858–0.9274	0.0321	0.3360	0.1407–0.8022	0.0140
SFA	0.9963	0.9893–1.0032	0.2937	0.9984	0.9913–1.0055	0.6595
SFT	0.9269	0.5335–1.6104	0.7876	1.2158	0.6989–2.1150	0.4891
SMA	1.0230	0.9906–1.0564	0.1657	1.0316	0.9985–1.0658	0.0616
SMI	1.0369	0.9588–1.1214	0.3644	1.0265	0.9506–1.1086	0.5041

**Table 3:** Detailed performance of the prediction models for MP classification in the validation dataset. LDA: Linear Discriminant Analysis, RF: Random Forest, MLP: Multi-Layer perceptron, NBB: Naïve Bayes Bernoulli, SVM: Support Vector Machine, LR: Logistic Regression.

Model	AUC	Threshold	95% CI	*p*	SEN	SPE	PPV	NPV
Clinical	0.608	N/A	0.515–0.696	reference	61.6%	60%	79.1%	38.9%
LDA Radiomics	0.695	0.8246	0.605–0.775	0.0902	80.2%	51.4%	80.2%	51.4%
RF Radiomics	0.662	0.8146	0.571–0.746	0.0308	43.0%	85.7%	88.1%	38.0%
MLP Radiomics	0.706	0.8019	0.616–0.785	0.1828	39.5%	94.3%	94.4%	38.8%
NBB Radiomics	0.716	0.9193	0.627–0.794	0.3556	48.8%	91.4%	93.3%	42.1%
SVM Radiomics	0.610	0.6636	0.517–0.697	0.0091	87.2%	37.1%	77.3%	54.2%
LR Radiomics	0.753	0.5604	0.667–0.827	reference	72.1%	80.0%	89.9%	53.8%
LDA Combined	0.748	0.9569	0.660–0.822	0.3723	57.0%	85.7%	90.7%	44.8%
RF Combined	0.681	0.7261	0.590–0.763	0.0340	65.1%	65.7%	82.4%	43.4%
MLP Combined	0.744	0.7562	0.657–0.819	0.3167	52.3%	88.6%	91.8%	43.1%
NBB Combined	0.743	0.5121	0.656–0.818	0.4226	67.4%	71.4%	85.3%	47.2%
SVM Combined	0.658	0.4808	0.566–0.742	0.0005	95.4%	28.6%	76.6%	71.4%
LR Combined	0.781	0.4998	0.697–0.851	reference	77.9%	74.3%	88.2%	57.8%

**Table 4:** Detailed performance of the prediction models for pCR classification in the validation dataset. LDA: Linear Discriminant Analysis, RF: Random Forest, MLP: Multi-Layer perceptron, NBB: Naïve Bayes Bernoulli, SVM: Support Vector Machine, LR: Logistic Regression.

Model	AUC	Threshold	95% CI	*p*	SEN	SPE	PPV	NPV
Clinical	0.626	N/A	0.534–0.713	reference	73.5%	51.7%	37.3%	83.3%
LDA Radiomics	0.771	0.2477	0.686–0.842	0.3984	58.8%	92.0%	74.1%	85.1%
RF Radiomics	0.708	0.4474	0.619–0.787	0.0333	52.9%	93.1%	75.0%	83.5%
MLP Radiomics	0.752	0.3331	0.665–0.826	0.2692	67.7%	85.1%	63.9%	87.1%
NBB Radiomics	0.767	0.4885	0.682–0.839	0.4112	64.7%	85.1%	62.9%	86.0%
SVM Radiomics	0.699	0.3596	0.609–0.779	0.0711	58.8%	86.2%	62.5%	84.3%
LR Radiomics	0.799	0.5044	0.716–0.866	reference	55.9%	93.1%	76.0%	84.4%
LDA Combined	0.817	0.6769	0.737–0.882	0.7568	61.8%	89.7%	70.0%	85.7%
RF Combined	0.774	0.3549	0.689–0.845	0.1062	61.8%	87.4%	65.6%	85.4%
MLP Combined	0.795	0.2064	0.712–0.863	0.3861	67.7%	88.5%	69.7%	87.5%
NBB Combined	0.803	0.3108	0.721–0.870	0.3216	73.5%	86.2%	67.6%	89.3%
SVM Combined	0.770	0.3704	0.684–0.841	0.1721	70.6%	82.8%	61.5%	87.8%
LR Combined	0.827	0.5001	0.747–0.889	reference	70.6%	85.1%	64.9%	88.1%

## Data Availability

The data that support the findings of this study are available from the corresponding author upon reasonable request.

## Supplementary Materials

The following supporting information can be downloaded at: https://www.mdpi.com/article/10.3390/bioengineering11070663/s1, Supplementary Data S1: Calibration analysis. Figure S1: The flowchart of feature selection. Figure S2: The flowchart of five-fold cross-validation. Figure from https://docs.ultralytics.com/guides/kfold-cross-validation/#introduction, (accessed on 19 May 2024). Figure S3: Selection of radiomics features using LASSO regression. (A) Selection of the optimal tuning parameter lambda for MP classification. (B) The coefficient profile plot of 10 MP-associated features against the optimal log(lambda) sequence. (C) Selection of the optimal tuning parameter lambda for pCR classification. (D) The coefficient profile plot of 9 pCR-associated features against the optimal log(lambda) sequence. Figure S4: Heatmap analysis of the selected radiomics features for MP and pCR classification. Table S1: Recent studies that constructed radiomic models to predict the efficacy of NAC in TNBC. Table S2: Comparison of the AUCs in the training dataset and validation dataset for the MP classification models. Table S3: Comparison of the AUCs in the training dataset and validation dataset for the pCR classification models. Table S4: Multiphase contrast-enhanced CT scan. Supplementary File S1: CT Scanning Parameters. Supplementary File S2: MRI Scanning Parameters for specific protocols. Supplementary File S3: Radiomics Features. Supplementary File S4: LASSO parameters. Supplementary File S5: Machine learning classifier parameters. Supplementary File S6: Feature weights and biological meaning.
