# Supplementary material for: Pretreatment Sarcopenia and MRI-Based Radiomics to Predict the Response of Neoadjuvant Chemotherapy in Triple-Negative Breast Cancer
Source: Bioengineering (Basel). 2024 Jun 28;11(7):663. doi: 10.3390/bioengineering11070663 (PMC11274092; doi:10.3390/bioengineering11070663)
Supplement: Supplementary file 1 [file bioengineering-11-00663-s001.zip › Supplementary Data S1.pdf]

Supplementary Data S1

Calibration analysis

For MP classification, the non-significant statistics of the LDA, RF, MLP, NBB, SVM, and LR classifier-based radiomics models were 0.695, 0.662, 0.706, 0.716, 0.610, and 0.753, respectively, and the non-significant statistics of the LDA, RF, MLP, NBB, SVM, and LR classifier-based combined models were 0.695, 0.662, 0.706, 0.716, 0.610, and 0.753, respectively. For pCR classification, the non-significant statistics of the LDA, RF, MLP, NBB, SVM, and LR classifier-based radiomics models were 0.695, 0.662, 0.706, 0.716, 0.610, and 0.753, respectively, and the non-significant statistics of the LDA, RF, MLP, NBB, SVM, and LR classifier-based combined models were 0.695, 0.662, 0.706, 0.716, 0.610, and 0.753, respectively. These results indicate that no significant deviations from an ideal fitting were found in all models.

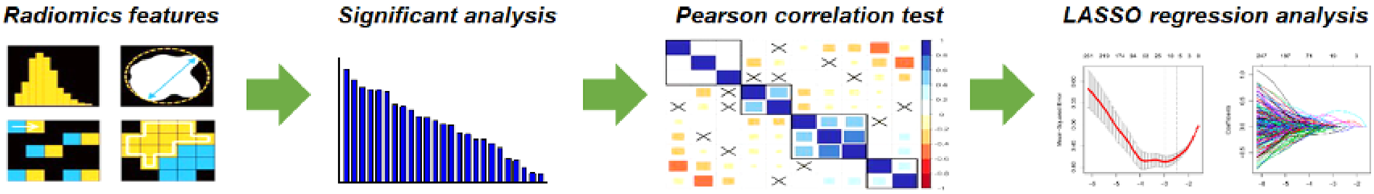

Figure S1. The flowchart of feature selection.

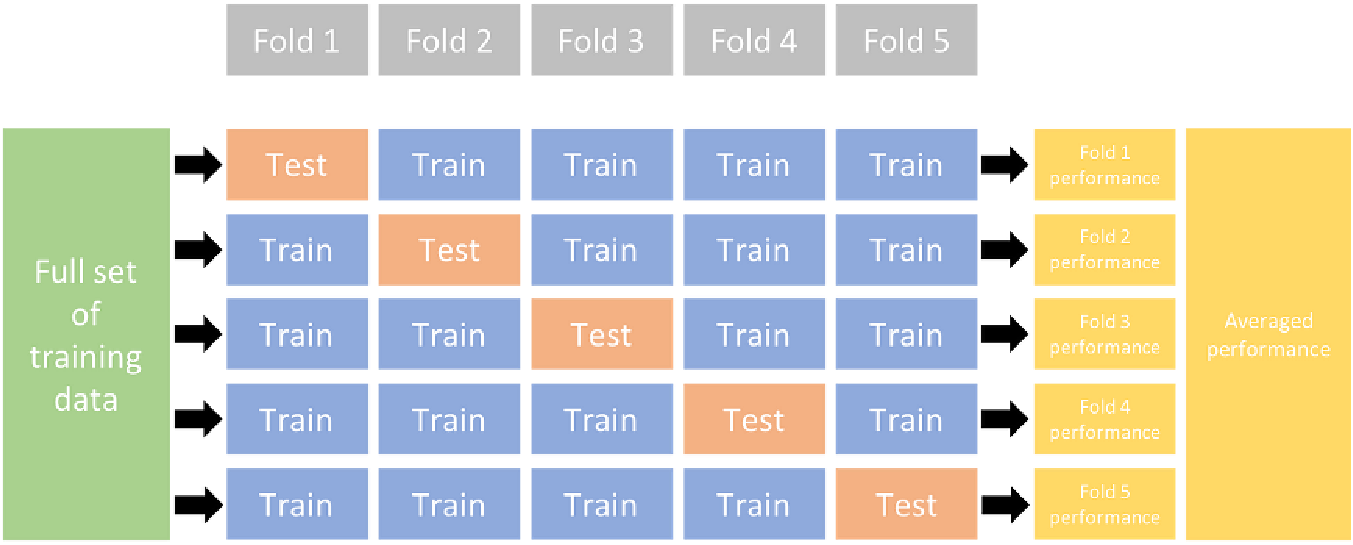

Figure S2. The flowchart of five-fold cross-validation. Figure from https://docs.ultralytics.com/guides/kfold-cross-validation/#introduction.

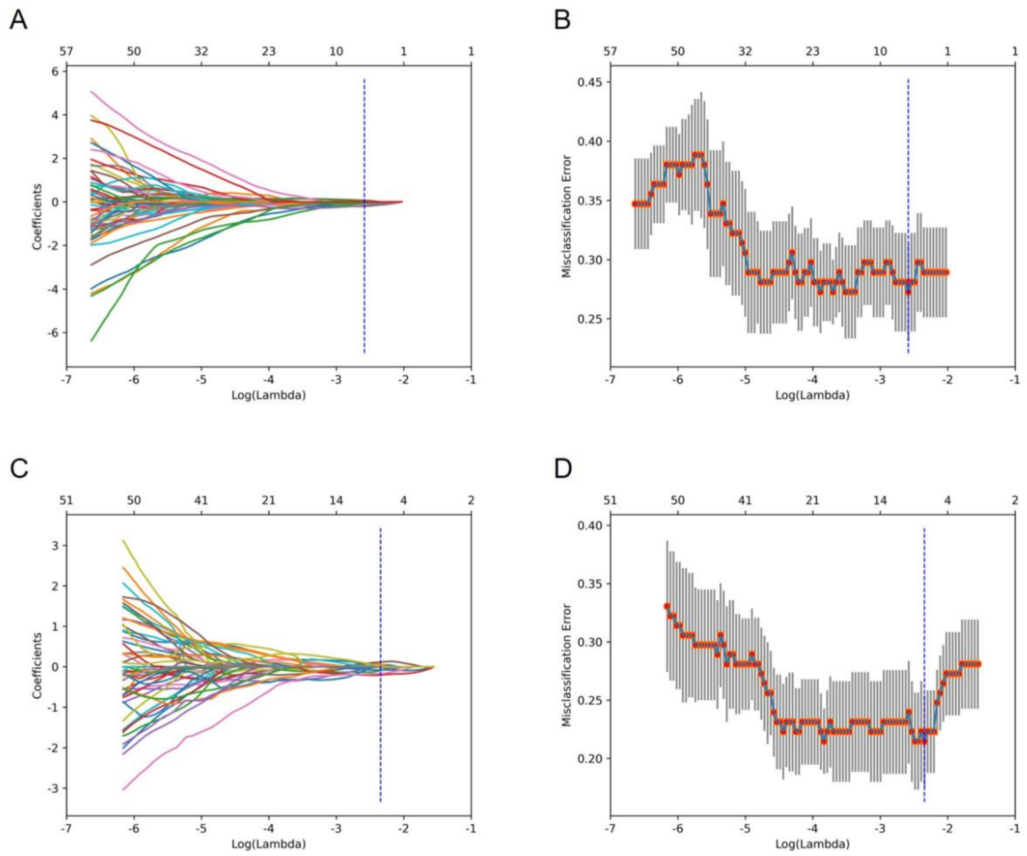

Figure S3. Selection of radiomics features using LASSO regression. (A) Selection of the optimal tuning parameter lambda for MP classification. (B) The coefficient profile plot of 10 MP-associated features against the optimal log(lambda) sequence. (C) Selection of the optimal tuning parameter lambda for pCR classification. (D) The coefficient profile plot of 9 pCR-associated features against the optimal log(lambda) sequence.

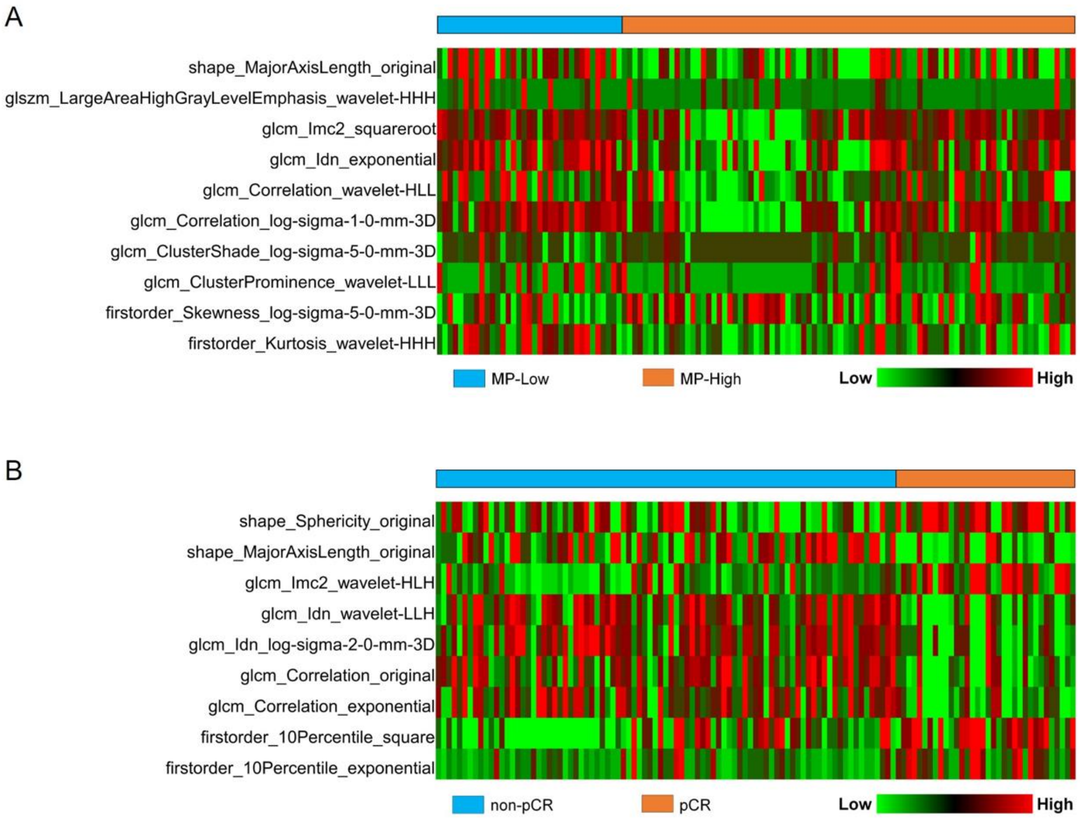

Figure S4. Heatmap analysis of the selected radiomics features for MP and pCR classification.

Table S1. Recent studies that constructed radiomic models to predict the efficacy of NAC in TNBC.

Purpose of the study	Methodologies	Predictive models	Model performance	Reference
Predicting pCR in TNBC	Quantitative radiomic features were extracted from contrast-enhanced MRI at baseline and after two cycles of treatment, and two radiomics-only models were constructed using light gradient boosting machine.	Radiomics-only models	Radiomics-only model AUCs of 0.71 and 0.73	19
	A radiogenomic model was developed by incorporating variant allele frequency features obtained from baseline core biopsies.	Radiogenomic model	Radiogenomic model AUC of 0.87	
Predicting pCR in TNBC	Constructed a stacked model integrating pre-, post-, and delta-models.	Multi-Layer Perception (MLP) model	MLP model AUC of 0.837-0.901	20
Predicting pCR in TNBC	Radiomics model was constructed using radiomics features taken from baseline MRI scans and manual tumor volumes of interest.	Radiomics model	Radiomics model AUC of 0.89	21
Predicting pCR in TNBC	Separate segmentation of tumor and parenchymal tissue and extraction of radiomics features on contrast-enhanced MRI images (T1-weighted, T2-weighted, diffusion-weighted, and dynamic contrast-enhanced images) at baseline to construct radiomics model.	Radiomics model	Radiomics model AUC of 0.83	22
Predicting the systemic recurrence of TNBC in patients after NAC	Three MRI radiomics models were constructed: a radiomics model based on pre-NAC MRI features (model 1), a radiomics model based on post-NAC MRI features (model 2), and a radiomics model based on pre- and post-NAC MRI features (model 3).	Radiomics models (Models 1, 2, and 3)	Models 1, 2, and 3 yielded AUCs of 0.814, 0.802, and 0.933	23
	A clinical model for predicting systemic recurrence was constructed using independent clinical predictors.	Clinical model	Clinical model AUC of 0.737	
Predicting disease-free survival (DFS) in TNBC patients	Development and validation of the intuitive nomogram to predict DFS based on radiomic features, MRI findings, and clinicopathologic variables.	A nomogram based on multifocal/centric disease status, pCR status, and Rad-score	The C-index of the model was 0.868	24

Model robustness evaluation

The AUCs between the training dataset and validation dataset of each model were compared by Delong's test. As shown in Supplementary Table 1 and Supplementary Table 2, only the LR classifier-based radiomics model for MP classification, the LR and NBB classifier-based combined model for MP classification, the LR and NBB classifier-based radiomics model for pCR classification, and the LR and NBB classifier-based combined model for pCR classification exhibited good robustness. Other models suffered from overfitting, as their AUCs in the training dataset were significantly higher than those in the validation dataset (all p-values < 0.05).

Table S2. Comparison of the AUCs in the training dataset and validation dataset for the MP classification models.

MP model	Classifier	Training dataset		Validation dataset		*p-value*
		AUC	95% CI	AUC	95% CI	
**Radiomics**	**LDA**	0.806	0.725-0.873	0.695	0.605-0.775	*<0.001*
	RF	0.969	0.921-0.992	0.662	0.571-0.746	*<0.001*
	MLP	0.875	0.803-0.928	0.706	0.616-0.785	*<0.001*
	NBB	0.770	0.685-0.842	0.716	0.627-0.794	*0.002*
	SVM	0.890	0.821-0.940	0.610	0.517-0.697	*<0.001*
	LR	0.780	0.696-0.851	0.753	0.667-0.827	*0.468*
Combined	LDA	0.865	0.791-0.921	0.748	0.660-0.822	*<0.001*
	RF	0.846	0.769-0.905	0.681	0.590-0.763	*<0.001*
	MLP	0.873	0.800-0.927	0.744	0.657-0.819	*<0.001*
	NBB	0.826	0.747-0.889	0.743	0.656-0.818	*0.055*
	SVM	0.995	0.961-1.000	0.658	0.566-0.742	*<0.001*
	LR	0.874	0.801-0.927	0.781	0.697-0.851	*0.086*

Table S3. Comparison of the AUCs in the training dataset and validation dataset for the pCR classification models.

pCR model	Classifier	Training dataset		Validation dataset		*p-value*
		AUC	95% CI	AUC	95% CI	
**Radiomics**	**LDA**	0.840	0.762-0.900	0.771	0.686-0.842	*<0.001*
	RF	0.985	0.944-0.998	0.708	0.619-0.787	*<0.001*
	MLP	0.877	0.805-0.930	0.752	0.665-0.826	*<0.001*
	NBB	0.817	0.737-0.882	0.767	0.682-0.839	*0.021*
	SVM	0.913	0.848-0.957	0.699	0.609-0.779	*<0.001*
	LR	0.819	0.738-0.883	0.799	0.716-0.866	*0.618*
Combined	LDA	0.888	0.818-0.938	0.817	0.737-0.882	*0.016*
	RF	0.999	0.967-1.000	0.774	0.689-0.845	*<0.001*
	MLP	0.969	0.920-0.992	0.795	0.712-0.863	*<0.001*
	NBB	0.838	0.760-0.899	0.803	0.721-0.870	*0.328*
	SVM	0.914	0.849-0.957	0.770	0.684-0.841	*<0.001*
	LR	0.840	0.762-0.900	0.827	0.747-0.889	*0.662*
