# Supplementary material for: Pretreatment Sarcopenia and MRI-Based Radiomics to Predict the Response of Neoadjuvant Chemotherapy in Triple-Negative Breast Cancer
Source: Bioengineering (Basel). 2024 Jun 28;11(7):663. doi: 10.3390/bioengineering11070663 (PMC11274092; doi:10.3390/bioengineering11070663)
Supplement: Supplementary file 1 [file bioengineering-11-00663-s001.zip › Supplementary File S1-Scanning Parameters.pdf]

Supplementary File S1

CECT Scanning Parameters

The contrast-enhanced CT images were acquired on various CT systems. All the patients underwent multiphase contrast-enhanced CT scans by using Omnipaque (350; GE Healthcare) contrast medium. Omnipaque was intravenously injected at a rate of 3 mL/s. The trigger threshold of the aorta reached 100 HU. Arterial phase (AP) and portal venous phase (PVP) were performed at 35 s, and 70 s, respectively, after intravenous injection of contrast. If the lesion showed continuous enhancement on PVP images, the delayed phase (120 s after intravenous injection of contrast) was added. Detailed scanning parameters are summarized in Table S4.

Table S4. Multiphase contrast-enhanced CT scan.

**Scan models**	**kV (voltage) pre/AP/PVP**	**Rotation time (s)**	**Single collimation width**	**Section thickness (mm) pre/AP/PVP**
Siemens Definition	120/120/120	0.5	1.2	7/5/5
Siemens SOMATOM Definition AS+	120/120/120	0.5	0.6	5/5/2
Siemens SOMATOM Definition Flash	100/120/120	0.5	0.6	5/2/2

AP: arterial phase; PVP: portal venous phase.
