# Supplementary material for: Pretreatment Sarcopenia and MRI-Based Radiomics to Predict the Response of Neoadjuvant Chemotherapy in Triple-Negative Breast Cancer
Source: Bioengineering (Basel). 2024 Jun 28;11(7):663. doi: 10.3390/bioengineering11070663 (PMC11274092; doi:10.3390/bioengineering11070663)
Supplement: Supplementary file 1 [file bioengineering-11-00663-s001.zip › Supplementary File S2-MRI Scanning Parameters.pdf]

Supplementary File S2

MRI Scanning Parameters

MR scanning parameters

The GE Discovery 750w 3.0T magnetic resonance scanner used a breast specific 8-channel coil, and the patient was in prone position with both breasts overhanging in the coil. The scanning sequence included:

1) Plain scan axial T2WI (TR 4000ms, TE 85ms, flip Angle 111°, matrix 320×256, FOV 320×320, slice thickness 4mm, slice spacing 1mm, excitation times 3, and fat suppression);
2) Plain scan axial T1WI (TR 400ms, TE 6.8-20.4ms, flip Angle 111°, matrix 320×256, FOV 320×320, slice thickness 4mm, slice spacing 1mm, excitation number 1, no fat-suppressed);
3) Axial DWI using EPI sequence (TR 4600ms, TE 76.9~18.3ms, flip Angle 90°, matrix 128×128, FOV 320×320, slice thickness 4mm, slice spacing 1mm, b value 0/1000s/mm^2, excitation number b=1000s/mm^2 =4, FOV 320×320, slice thickness 4mm, slice spacing 1mm; Excitation times b=0s/mm^2 =1, and fat suppression).
4) Coronal T2 sequence (TR 4000ms, TE 85ms, flip Angle 111°, matrix 320×256, FOV 320×320, slice thickness 4mm, slice spacing 1mm, excitation times 3, and fat suppression);
5) Axial T1 dynamic contrast-enhanced scan (scan 1+5 phase, each phase scan time 2min, TR 4.3ms, TE 2.1ms, flip Angle 10°, matrix 320×256, FOV 320×320, slice thickness 1mm, slice spacing 0, and fat suppression). Gadolinium contrast agent was used for intravenous injection through a dual-syringe high-pressure syringe at a flow rate of 3ml/s, and the injection dose was calculated as 0.2ml/kg, followed by a bolus of 20ml normal saline at a flow rate of 5ml/s.

After the dynamic contrast-enhanced scan, oblique sagittal T1 spoiled gradient recalled echo sequence scan was performed in the bilateral axillary region at about 45°. Then, the data of 6 phases of dynamic contrast-enhanced scan were imported into GE ADW 4.6 postprocessing workstation for analysis. The area with obvious enhancement of the lesion was selected, the ROI was drawn, and the time intensity curve (TIC) was obtained.
