# Supplementary material for: Pretreatment Sarcopenia and MRI-Based Radiomics to Predict the Response of Neoadjuvant Chemotherapy in Triple-Negative Breast Cancer
Source: Bioengineering (Basel). 2024 Jun 28;11(7):663. doi: 10.3390/bioengineering11070663 (PMC11274092; doi:10.3390/bioengineering11070663)
Supplement: Supplementary file 1 [file bioengineering-11-00663-s001.zip › Supplementary File S4-LASSO parameters.pdf]

Supplementary File S4

LASSO parameters

alpha = 1.0; fit_intercept = True; normalize = 'deprecated'; precompute = False; copy_X = True; max_iter = 1000; tol = 0.0001; warm_start = False; positive = False; random_state = None; selection (default='cyclic'); type.measure = mean squared error
